# Construction and validation of a nomogram for predicting the prognosis of patients with lymph node-positive invasive micropapillary carcinoma of the breast: based on SEER database and external validation cohort

**DOI:** 10.3389/fonc.2023.1231302

**Published:** 2023-10-24

**Authors:** Yifei Li, Jinzhao Liu, Zihang Xu, Jiuyan Shang, Si Wu, Meng Zhang, Yueping Liu

**Affiliations:** ^1^ Department of Pathology, The Fourth Hospital of Hebei Medical University, Shijiazhuang, China; ^2^ The Second Department of Thyroid and Breast Surgery, Cangzhou Central Hospital, Cangzhou, China; ^3^ College of Basic Medical Sciences, Hebei Medical University, Shijiazhuang, China

**Keywords:** IMPC, LNM, prognosis, nomogram, SEER database

## Abstract

**Background:**

Invasive micropapillary carcinoma (IMPC) of the breast is a rare subtype of breast cancer with high incidence of aggressive clinical behavior, lymph node metastasis (LNM) and poor prognosis. In the present study, using the Surveillance, Epidemiology, and End Results (SEER) database, we analyzed the clinicopathological characteristics and prognostic factors of IMPC with LNM, and constructed a prognostic nomogram.

**Methods:**

We retrospectively analyzed data for 487 breast IMPC patients with LNM in the SEER database from January 2010 to December 2015, and randomly divided these patients into a training cohort (70%) and an internal validation cohort (30%) for the construction and internal validation of the nomogram, respectively. In addition, 248 patients diagnosed with IMPC and LNM at the Fourth Hospital of Hebei Medical University from January 2010 to December 2019 were collected as an external validation cohort. Lasso regression, along with Cox regression, was used to screen risk factors. Further more, the discrimination, calibration, and clinical utility of the nomogram were assessed based on the consistency index (C-index), time-dependent receiver operating characteristic (ROC), calibration curve, and decision curve analysis (DCA).

**Results:**

In summary, we identified six variables including molecular subtype of breast cancer, first malignant primary indicator, tumor grade, AJCC stage, radiotherapy and chemotherapy were independent prognostic factors in predicting the prognosis of IMPC patients with LNM (*P* < 0.05). Based on these factors, a nomogram was constructed for predicting 3- and 5-year overall survival (OS) of patients. The nomogram achieved a C-index of 0.789 (95%CI: 0.759-0.819) in the training cohort, 0.775 (95%CI: 0.731-0.819) in the internal validation cohort, and 0.788 (95%CI: 0.756-0.820) in the external validation cohort. According to the calculated patient risk score, the patients were divided into a high-risk group and a low-risk group, which showed a significant difference in the survival prognosis of the two groups (*P*<0.0001). The time-dependent ROC curves, calibration curves and DCA curves proved the superiority of the nomogram.

**Conclusions:**

We have successfully constructed a nomogram that could predict 3- and 5-year OS of IMPC patients with LNM and may assist clinicians in decision-making and personalized treatment planning.

## Introduction

**Figure 1:**
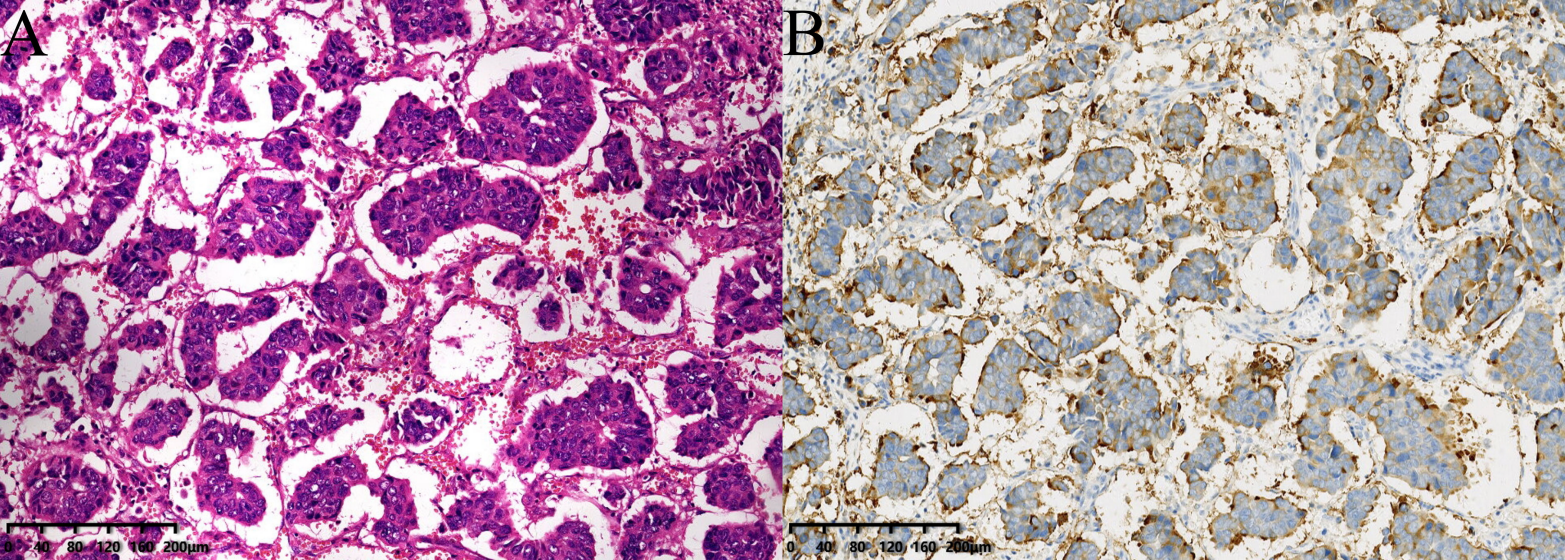
**(A)** The tumor cells of IMPC are arranged in a pseudopapillary structure without fiber vascular axis, and there are irregular and narrow interstitial structures between the cancer cell cluster and the surrounding stroma (stained with hematoxylin and eosin (HE) 100×). **(B)** Epithelial membrane antigen (EMA) staining shows staining positive sites are located on the mesenchymal side of the cancer cell mass at the edge of the cell membrane and interstitial lumen, which is characteristics of polarity reversal (100×).

Invasive micropapillary carcinoma of breast (IMPC) is a special type of invasive breast cancer, accounting for 0.9% - 8% of all breast cancer (1, 2). The tumor cells of IMPC are arranged in a pseudopapillary structure without a fibrous vascular axis. Epithelial membrane antigen (EMA) immunohistochemistry confirmed the reverse polarity of the neoplastic cells, and there is an irregular narrow gap structure between the cancer cell cluster and the surrounding stroma (**Figure 1**). Previous studies have considered that the morphological characteristics of IMPC are related to tumor biological behavior, particularly to tumor invasion, metastasis, and prognosis (3, 4). Even if the proportion of micropapillary structures is less than 10%, compared with breast cancer of the same pathological type without micropapillary components, the invasive capacity of cancer is also significantly higher (5). Compared with invasive ductal carcinoma of no special type (IDC NST), IMPC is prone to local recurrence, distant metastasis, and lymph node metastasis (LNM), with a high incidence of LNM of 44% -85% (6). 24.9% of patients are accompanied by lymph node invasion at the primary diagnosis (3). Lymph node status is not only an important basis for breast cancer staging, but also an independent prognostic indicator of breast cancer (7). Research has shown that breast IMPC patients with LNM have a higher risk of recurrence and a poorer prognosis (8). In a population-based study by Chen et al. (9), 52.9% of breast IMPC patients had LNM, with a 5-year overall survival (OS) only 83.8%.

The American Joint Committee for Cancer (AJCC) staging system is a widely used tool for clinicians to predict disease outcomes and guide therapeutic decision making (10). However, this staging only includes anatomical factors and does not cover factors such as cancer biology and treatment, which is insufficient to accurately predict the prognosis of all IMPC patients. Due to the limited benefits of neoadjuvant chemotherapy for lymph node-positive IMPC patients, using a unified predictive model to predict the survival of IMPC patients inevitably leads to erroneous estimates of survival. In the era of precision medicine, the application of nomograms in individualized risk prediction is well recognized in a wide variety of cancers. Although there are currently various nomograms to predict the prognosis of IMPC, there is still a lack of nomogram to predict the prognosis of IMPC patients with LNM. Given the crucial role of powerful prognostic prediction tools in determining appropriate treatment methods to improve survival, it is necessary to discuss and construct predictive models for IMPC patients with positive lymph nodes to improve the accuracy of survival prediction. Compared with previous studies (11, 12), in order to avoid redundancy or overfitting, our study used LASSO regression to screen for significant factors related to OS and construct a nomogram. In addition, we not only built a network calculator based on the nomogram, but also conducted risk stratification, creating more convenience for clinical practice. Finally, we validated the predictive performance of the developed nomogram internally and obtained validation from the largest external cohort in China.

Therefore, the aim of this study is to construct and validate a nomogram based on clinicopathological characteristics to predict the prognosis of IMPC patients with LNM. The nomogram is used to divide patients into high-risk and low-risk groups, which has important clinical guiding significance in improving accurate staging, adjuvant treatment strategies and prognosis evaluation.

## Materials and methods

### Data sources and patient selection

The Surveillance, Epidemiology, and End Results (SEER) database is a cancer database in the United States that includes 18 cancer registries, covering 47.9% of the US population (13). In this study, we used SEER * Stat 8.4.0 software to extract clinical pathological data and prognosis results of patients from the SEER 18 registration database. All patients we selected were lymph node-positive IMPC patients (n=495) from January 2010 to December 2015. In the process of data screening, we found that missing values and outliers (n=8) accounted for a small proportion of the total number of samples (495). Therefore, the missing data and outliers were removed to improve the confidence of the statistical results. The final sample (n=487) with complete clinical-pathological characteristics and follow-up data is used for subsequent analysis. According to previous studies, these patients were randomly divided into a training cohort (70%) and an internal validation cohort (30%), respectively, for the construction and validation of nomogram (14, 15). We consider 7:3 to be an appropriate ratio to apply to this study. Using most of the data to construct a column chart can ensure the accuracy of the model, while a smaller portion of the data is used for validation to prevent overfitting. In addition, we collected all IMPC patients with positive lymph nodes who visited the Fourth Hospital of Hebei Medical University from January 2010 to December 2019 as external validation cohorts to further validate the constructed nomogram(n=248).

Eligible patients were determined based on the following inclusion criteria: 1) IMPC confirmed by pathology; 2) Women with a diagnosis age of ≥ 18 years old; 3) Patients with pathologically confirmed lymph node metastases; 4) Received surgical treatment. The exclusion criteria are as follows: 1) Distant metastasis; 2) Bilateral breast cancer; 3) Lack of clinical-pathological characteristics and follow-up data.

There was no requirement for ethical approval since all of the data from the SEER database was obtained in a public method. The study was approved by the Ethics Review Committee of the Fourth Hospital of Hebei Medical University (Approval Number:2020K-1334) and received written informed consent from all participants.

### Variable collection

We recorded the following patient information: baseline demographics (age, race, marital status), tumor characteristics (laterality, location, size, first malignant primary indicator, histological grade, tumor node metastasis (TNM) staging, estrogen receptor (ER), progesterone receptor (PR), human epidermal growth factor receptor 2 (HER2), molecular typing, number of positive lymph nodes, etc.), treatment information (surgical methods, radiotherapy, and chemotherapy), and survival outcomes (survival status, survival time). We restaged all patients in the study under the AJCC 8th edition stage group (16). In the analysis, some continuous variables were converted into categorical variable, such as age, tumor size, and number of positive lymph nodes. Patients were divided into two groups by age at diagnosis (<50 and ≥ 50); tumor size was divided into three groups (<2cm, ≥ 2cm and<5cm and ≥ 5cm) and the number of lymph node metastases was divided into two groups: 1-3 and ≥ 4. The endpoint of this study was OS, which was defined as the time from surgery to the date of last follow-up or death from any cause. The patients in external validation cohort were followed up by means of inpatient medical record review, outpatient follow-up, and telephone. The last follow-up date was November 10, 2022.

### Statistical analysis

The raw data of training cohort were preprocessed by Z-score normalization and the same preprocessing procedure was applied to the validation cohort (**Supplementary Figures 1**, **2**). The LASSO regression algorithm was used to screen clinicopathological characteristics that were significantly correlated with prognosis. Then, based on the final results of LASSO regression, the independent prognostic factors for OS were identified using a multivariate Cox regression analysis in the training cohort, and the hazard ratio (HR) and 95% confidence interval (95% CI) of these variables were calculated. Based on these independent prognostic factors, a nomogram was constructed to predict the 3- and 5-year OS of IMPC patients with positive lymph nodes. Internal and external validation was also performed to further evaluate the nomogram model. The consistency index (C-index), time-dependent receiver operating characteristic (ROC), time-dependent area under the ROC curve (AUC) were used to evaluate the discrimination ability. The AUC or C-index ranged from 0.5 to 1.0, with 0.5 indicating the random chance of the model correctly predicting outcomes and 1.0 indicating perfect predictive performance. Usually, C-index and AUC value >0.7 indicate the satisfactory discriminative ability of the predictive tool. Calibration curves were plotted to assess the calibration ability of the nomogram. A calibration curve was constructed using the bootstrap method (1000 cycles) to show the deviation between the predicted value and the actual probability of occurrence. The standard curve is a straight line passing through the origin of the coordinate axis with a slope of 1. If the predicted calibration curve is closer to the standard curve, the better the prediction ability of the nomogram. To compare the accuracy of the new model with that of the traditional AJCC staging model, the net reclassification improvement (NRI) and the integrated discrimination improvement (IDI) were determined. The clinical application value of the nomogram was evaluated using decision curve analysis (DCA). In addition, we divided all patients into high-risk and low-risk groups according to their risk values on the nomogram. The log rank test was calculated to compare the survival difference between two groups and Kaplan-Meier curves were used to visualize the results.

Continuous variables are described by mean ± standard deviation, and categorical variables are expressed as numbers (percentage). All statistical analyses were conducted using R software (version 4.1.1; http://www.Rproject.org). All statistical tests are bilateral, and a *P* value < 0.05 would be considered statistically significant.

## Results

### Baseline characteristics of patients

**Figure 2:**
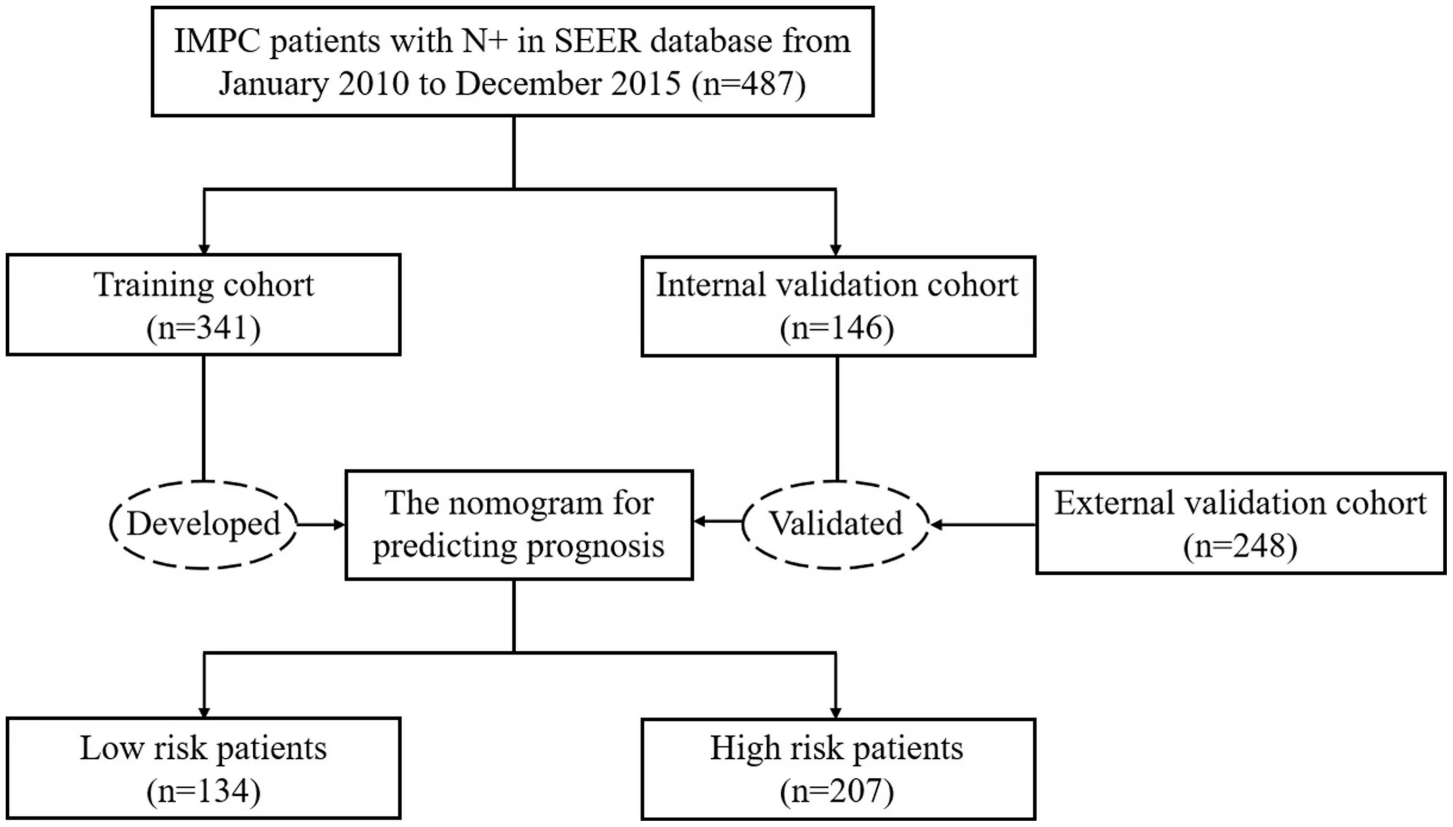
Study flow chart of IMPC patients with LNM.

According to the inclusion and exclusion criteria, a total of 487 IMPC patients were collected from the SEER database and randomly assigned to a training cohort (n=341) and a internal validation cohort (n=146) at a 7:3 ratio. In addition, we employed an external validation cohort composed of 248 Chinese patients who received treatment at the Fourth Hospital of Hebei Medical University from January 2010 to December 2019 using the same patient selection criteria as mentioned above. The study flow chart was shown in **Figure 2** and baseline characteristics of the enrolled patients were summarized in **Table 1**. There were no statistically significant differences in clinicopathological characteristics between the training and internal validation cohorts (*P* > 0.05).

**Table 1:** Clinicopathological characteristics of IMPC patients in training, internal validation, and external validation cohorts .

Variables	SEER cohort			China cohort
	Training cohort n=341 (%)	Internal validation cohort n=146 (%)	*P*	External validation cohort n=248 (%)
Age			0.57	
<50	95(27.9)	37(25.3)		100(40.3)
≥50	246(72.1)	109(74.7)		148(59.7)
Marital status			0.30	
Single	67(19.6)	19(13.0)		8(3.2)
Married	256(75.2)	123(84.3)		240(96.8)
Unknown	18(5.2)	4(2.7)		0(0.0)
Race			0.28	
Asian or Pacific Islander	25(7.3)	19(13.0)		248(100.0)
White	255(74.8)	101(69.2)		0(0.0)
Black	59(17.3)	26(17.8)		0(0.0)
American Indian/Alaska Native	1(0.3)	0(0.0)		0(0.0)
Unknown	1(0.3)	0(0.0)		0(0.0)
First primary malignancy			0.73	
No	46(13.5)	18(12.3)		20(8.1)
Yes	295(86.5)	128(87.7)		228(91.9)
Primary laterality			0.85	
Left	162(48)	68(46.6)		123(49.6)
Right	179(52)	78(53.4)		125(50.4)
Primary site			0.18	
Center	19(5.6)	13(8.9)		17(6.9)
Upper inner	26(7.6)	19(13.0)		48(19.4)
Lower inner	21(6.1)	10(6.8)		19(7.7)
Upper outer	118(34.6)	40(27.4)		66(26.6)
Lower outer	27(7.9)	11(7.5)		25(10.1)
Other	130(38.2)	53(36.3)		73(29.4)
Tumor size			0.37	
<2cm	117(34.3)	40(27.4)		56(22.6)
≥2cm <5cm	140(41.1)	71(48.6)		180(72.6)
≥5cm	84(24.6)	35(24.0)		12(4.8)
Regional nodes positive			0.83	
1-3	201(58.9)	88(60.3)		111(44.8)
≥4	140(41.1)	58(39.7)		137(55.2)
T stage (AJCC-8th)			0.34	
T1	127(37.2)	47(32.2)		95(38.3)
T2	136(40.0)	63(43.2)		116(46.8)
T3	53(15.5)	24(16.4)		22(8.9)
T4	25(7.3)	12(8.2)		15(6.0)
N stage (AJCC-8th)			0.55	
N1	219(64.2)	97(66.4)		162(65.3)
N2	63(18.5)	28(19.2)		46(18.5)
N3	59(17.3)	21(14.4)		40(16.1)
TNM stage (AJCC-8th)			0.96	
I	33(9.7)	7(4.8)		26(10.5)
II	150(44.0)	76(52.1)		137(55.2)
III	158(46.3)	63(43.2)		85(34.3)
Histologic grade			0.20	
I	22(6.5)	5(3.4)		27(10.9)
II	171(50.1)	70(47.9)		47(19.0)
III	137(40.2)	66(45.2)		154(62.1)
Unknown	11(3.2)	5(3.4)		20(8.1)
ER status			0.24	
Negative	29(8.5)	16(11.0)		10(4.0)
Positive	305(89.4)	129(88.4)		235(94.8)
Unknown	7(2.1)	1(0.7)		3(1.2)
PR status			0.30	
Negative	56(16.4)	28(19.2)		19(7.7)
Positive	277(81.3)	117(80.1)		226(91.1)
Unknown	8(2.3)	1(0.7)		3(1.2)
HER2 status			0.69	
Negative	250(73.3)	109(74.7)		179(72.2)
Positive	76(22.3)	33(22.6)		66(26.6)
Unknown	15(4.4)	4(2.7)		3(1.2)
Molecular subtypes			0.81	
Luminal A	230(67.4)	100(68.5)		139(56.0)
Luminal B	65(19.1)	26(17.8)		69(27.8)
HER2 enriched	14(4.1)	6(4.1)		17(6.9)
Triple negative	12(3.5)	100(68.5)		11(4.4)
Unknown	20(5.9)	4(2.7)		12(4.8)
Surgery			0.71	
BCS	127(37.2)	51(34.9)		82(33.1)
Mastectomy	198(58.1)	89(61.0)		166(66.9)
No	16(4.7)	6(4.1)		0(0.0)
Radiotherapy			0.46	
No/unknown	121(35.5)	57(39.0)		114(46.0)
Yes	220(64.5)	89(61.0)		134(54.0)
Chemotherapy			0.86	
No/unknown	82(24.0)	34(23.3)		138(55.6)
Yes	259(76.0)	112(76.7)		110(44.4)

IMPC, Invasive Micropapillary Carcinoma; AJCC, American Joint Committee on Cancer; ER, Estrogen Receptor; PR, Progesterone Receptor; HER2, Human Epidermal Growth Factor Receptor 2; BCS, Breast conserving surgery.

### Construction of nomogram

**Figure 3:**
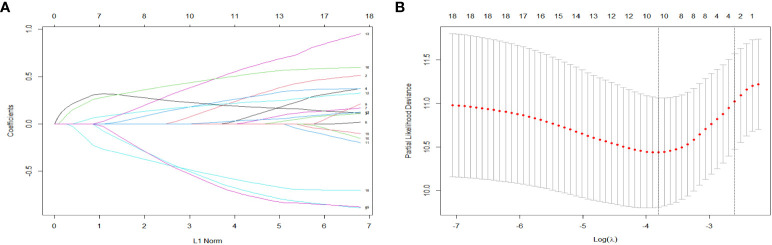
LASSO coefficient distribution of predictive factors **(A)** and selection of the optimal parameter (lambda) in the LASSO model **(B)**.

**Figure 4:**
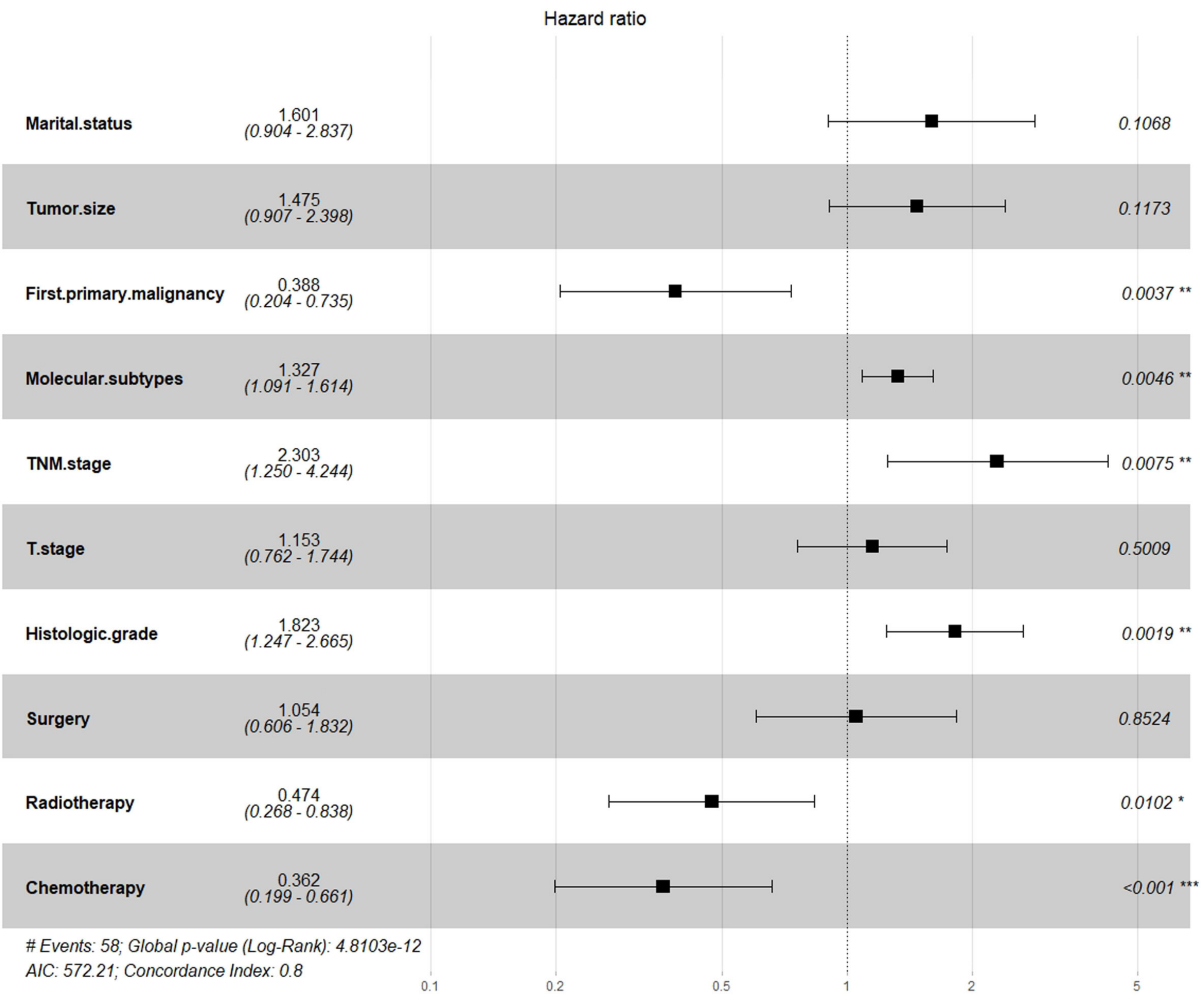
Multifactor Cox regression analysis forest map.

A total of 19 related variables in our study were originally input into the LASSO regression method by 10-fold cross validation to determine the prognostic factors of 3- and 5-year overall survival (OS) in breast IMPC patients with LNM. Optimized lambda determined in LASSO regression model, with min lambda 0.04532564, there were 10 indexes selected: marital status, whether it is the first malignant primary indicator, tumor size, clinical T stage, TNM stage, tumor grade, molecular subtype of breast cancer, operation mode, whether it receives chemotherapy and radiotherapy (**Figure 3**). Then, variables selected by LASSO regression were included in the multivariable Cox regression analysis, and the results were presented as HR and 95% CI. The following factors are significantly related to the prognosis of patients: whether it is the first malignant primary indicator (HR=0.40, 95% CI=0.21-0.76, *P*=0.005), TNM stage (HR*=*2.32, 95% CI=1.26-4.26, *P*=0.007), tumor grade (HR=1.80, 95% CI=1.23-2.64, *P*=0.002), molecular subtype of breast cancer (HR=1.33, 95% CI=1.10-1.62, *P*=0.009), whether it receives chemotherapy (HR=0.35, 95% CI=0.19-0.64, *P*<0.001), and whether it receives radiotherapy (HR=0.48, 95% CI=0.27-0.86, *P*=0.013) (**Figure 4**).

**Figure 5:**
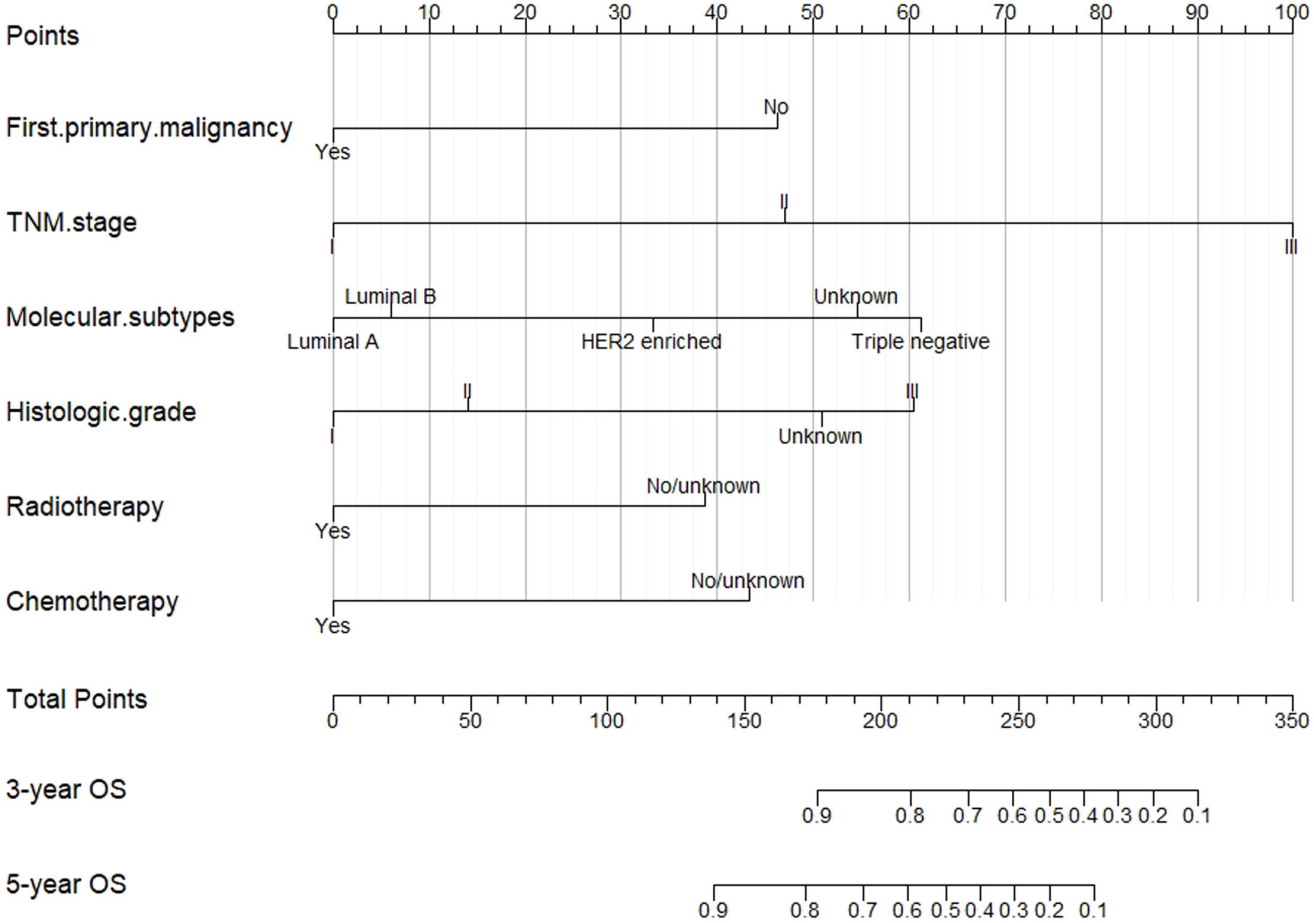
**A** nomogram for predicting 3- and 5-year OS of IMPC patients with LNM.

On the basis of multivariate Cox regression in the training cohort, a nomogram that integrated six independent risk factors was established to predict 3-year and 5-year OS in lymph node-positive IMPC patients (**Figure 5**). The value of each risk factor is assigned a score on the point scale axis. A total score could be easily calculated by adding each single score and located this sum on the total point scale axis. The probability of 3-year and 5-year OS can be estimated by calculating the total number of points from the vertical line of the variable to the point axis. The breast IMPC prognosis nomogram established by this research institute can be obtained through https://liyifei-1996.shinyapps.io/IMPCDynNomapp/ access and use online.

### Evaluation and validation of the nomogram

**Figure 6:**
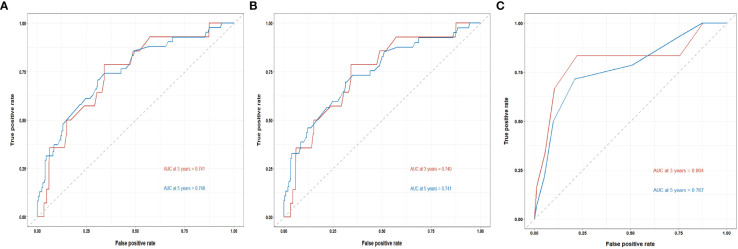
Time-dependent ROC curves for the nomogram’s prediction of 3-, and 5-year OS in the training cohort **(A)**, internal validation cohort **(B)**, and external validation cohort **(C)**.

**Figure 7:**
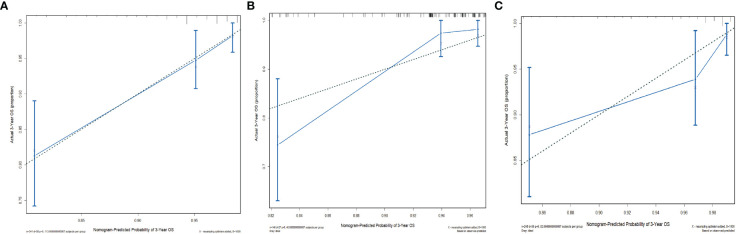
Calibration curves for the nomogram’s prediction of 3-year OS in the training cohort **(A)**, internal validation cohort **(B)**, and external validation cohort **(C)**.

**Figure 8:**
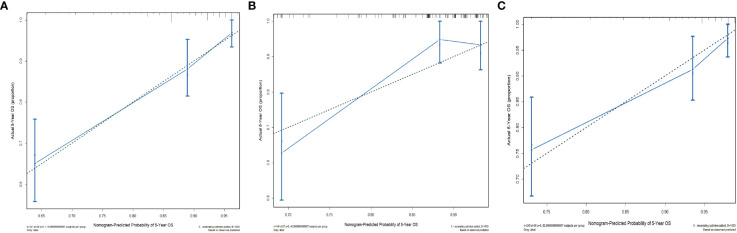
Calibration curves for the nomogram’s prediction of 5-year OS in the training cohort **(A)**, internal validation cohort **(B)**, and external validation cohort **(C)**.

**Figure 9:**
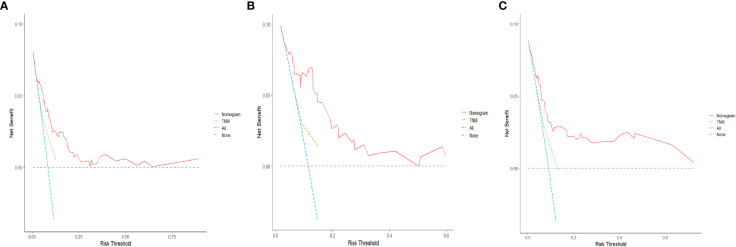
DCA curves for the nomogram’s prediction of 3-year OS in the training cohort **(A)**, internal validation cohort **(B)**, and external validation cohort **(C)**.

**Figure 10:**
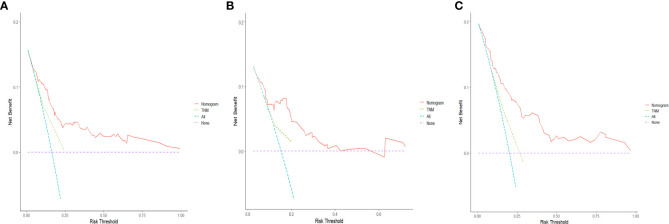
DCA curves for the nomogram's prediction of 5-year OS in the training cohort **(A)**, internal validation cohort **(B)**, and external validation cohort **(C)**.

The C-indices of the training cohort, internal validation cohort, and external validation cohort are 0.789 (95% CI: 0.759-0.819), 0.775 (95% CI: 0.731-0.819), and 0.788 (95% CI: 0.756-0.820), respectively. The time-dependent ROC curves show that the nomogram has good predictive performance for 3-year and 5-year OS in breast IMPC patients with LNM (**Figure 6**). As illustrated in **Figure 6**, the AUCs of 3- and 5-year OS for the training cohort are 0.741 and 0.748, respectively; meanwhile, the corresponding values for the internal validation cohort are 0.740 and 0.741, respectively; and 0.804 and 0.767 in the external validation cohort. The calibration curves of the training cohort, internal validation cohort, and external validation cohort indicate that the prediction probability of the nomogram is close to the actual observation probability, showing a strong consistency (**Figures 7**, **8**). As a novel method for evaluating diagnostic and prognostic prediction models, DCA curves are also drawn to evaluate the clinical application value of the nomogram which show that compared with the traditional TNM staging method, the nomogram could more accurately predict the OS of IMPC patients at 3- and 5 years (**Figures 9**, **10**).

### Ability of nomogram to stratify patient risk

**Figure 11:**
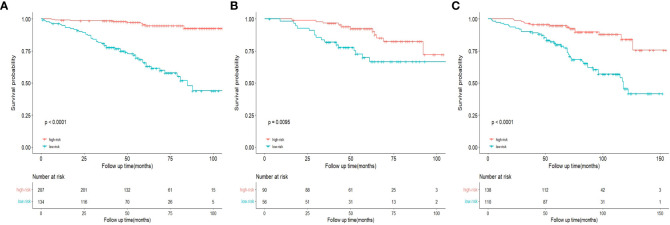
Kaplan-Meier curves of OS for risk stratification in the training cohort **(A)**, internal validation cohort **(B)**, and the external validation cohort **(C)**.

Based on the prognostic signature, we calculate the risk score for each patient and stratified all patients into a high-risk group (score≥152.884) or a low-risk group (score<152.884). Compared with the low-risk group, OS is significantly lower in patients with breast cancer in the high-risk group (**Figure 11A**). In addition, the Kaplan-Meier curves of the internal and the external validation cohort show similar performances to those of the training cohort, demonstrating the significant difference in survival prognoses between the predicted high- and low-risk groups (**Figures 11B, C**).

## Discussion

IMPC is a special type of breast cancer with poor prognosis. Although recent studies have shown no statistical differences between IMPC and IDC-NST in OS and DFS (17, 18), due to its unique morphological structure and invasive biological behavior, most IMPC patients are more likely to receive intensive treatment in clinical decision-making. Therefore, an accurate risk model can guide clinicians to identify high-risk patients and formulate more personalized treatment plans for IMPC patients. As far as we know, this is the first study to construct a nomogram integrated clinicopathological characteristics for predicting the prognosis of IMPC patients with LNM. Our model has higher C-index in the training cohort and external validation cohort than the nomogram previously published by Chen et al. (11) (training cohort C-index: 0.789 vs 0.756, external validation cohort C-index: 0.788 vs 0.742), and a higher AUC value in the external validation cohort (3-year OS: 0.804 vs 0.766, 5-year OS: 0.767 vs 0.725), indicating that the nomogram has higher accuracy in predicting patient prognosis. In the training cohort and two validation cohorts, the calibration curves also showed a high degree of agreement between predicted and actual observed results, reflecting the reliability of prediction models. Further DCA analysis also demonstrated that our nomogram has promising clinical applicability compared to the traditional AJCC staging system. In addition, the risk stratification model based on this nomogram can effectively classify patients into high-risk and low-risk groups and OS can be distinguished. Patients in the low-risk group may had a good prognosis, while for patients with higher risk, clinicians can make treatment interventions and treatment plan adjustments in a timelier manner to improve the prognosis.

This study included 487 lymph node-positive IMPC patients from the SEER database and 248 patients from the Fourth Hospital of Hebei Medical University. The SEER database is the most comprehensive database including sociodemographic data, treatment history, clinical pathological and molecular factors, allowed us to adjust for a high number of important confounders and the interaction between them (19). And compared with the nomogram published by Wang et al. (12), we established an external validation dataset from different races, regions, and economic and social environment populations. The nomogram achieves good accuracy and stability in internal and external validation, and has applicability in various clinical scenarios. To avoid overfitting or underfitting the model, we tried to determine the optimal model using LASSO regression and Cox regression (20). The former can effectively screen variables, while the latter can be used to modeled and visualized for direct interpretation. Chen et al. (11) and Wang et al. (12) had established a nomogram for predicting the prognosis of IMPC patients through univariate and multivariate Cox analysis, respectively. However, we considered that too many predictors are unnecessary for clinical application and the inclusion of variables that are not significantly related to the outcome contributed little to the improvement of the model. Compared with the traditional multivariable regression, LASSO regression is considered as a better method to select variables since it can minimize overfitting and reduce the complexity of the model by using a loss function or penalty term that is added to the objective function (21–23). In addition, we have developed a network calculator based on the nomogram. By inputting patient prognosis related information, it is easier and more intuitive to predict the OS of lymph node-positive IMPC patients, which is of great significance for guiding individualized comprehensive treatment and improving patient survival.

Our study determined six variables including molecular subtype of breast cancer, first malignant primary indicator, tumor grade, AJCC stage, radiotherapy and chemotherapy are independent risk factors for OS in breast IMPC patients with positive lymph nodes. Compared to the not routinely measured and costly molecular markers, these variables have advantages in convenience, easy access and low cost, which will improve the follow-up compliance and the survival rate of patients. Traditionally, histological grade and AJCC stage are key factors for the prognosis of breast cancer patients. The higher the histological grade and AJCC stage, the worse the prognosis of IMPC patients (24, 25). A sizeable study of 1268 patients suggested that pathologic data (i.e., grade/stage) was sufficient to replace the use of the Oncotype RS distinguish between low-risk and high-risk populations. Our model also indicates that AJCC stage contributes the most to the prognosis, followed by histological grade. The inclusion of additional information regarding clinicopathological characteristics provides our nomograms with a more accurate prognosis prediction ability, which can be used to improve the AJCC TNM staging system or as a supplementary version. The currently accepted classical classification of breast cancer uses microarray-based breast cancer tumor gene expression profiles to classify breast cancer into four intrinsic subtypes: Triple negative, HER2 enriched, Luminal A and Luminal B (26). Consistent with previous studies (27–29), we prove that molecular subtypes demonstrate a strong correlation to breast cancer prognosis. Luminal A and B subtypes have a relatively good prognosis; however, Triple negative tumors and HER2 tumors have very poor prognosis. Based on the specific molecular features of the tumor, clinicians can provide appropriate strategy for follow-up and treatment (30). In clinical practice, surgery combined with radiotherapy and chemotherapy is currently an important part of the standardized treatment system for breast cancer, and active treatment is of great clinical significance in improving the quality of life and prolonging the survival time for patients (31–33). Our research also confirmed that radiotherapy and chemotherapy are important factors affecting the prognosis of breast cancer. In the analysis of prognostic factors, we also found that patients whose breast cancer was not the first primary malignancy tended to have a poorer prognosis, which is also true in other cancers (34–36). In general, higher number of positive lymph nodes and lymph node metastasis rate are associated with poor prognosis of breast cancer patients (37–39). For IMPC patients, a literature (15) shows through univariate analysis that IMPC patients with ≥4 positive lymph nodes have shorter OS compared to lymph node negative patients, while the OS of IMPC patients with 1-3 positive lymph nodes is similar to that of patients with lymph node negative diseases. Our study also found that the number of positive lymph nodes has no impact on the prognosis of IMPC patients with LNM. This finding may be unique to this particular subtype of breast cancer, although several contributing and confounding factors may also play a role. In addition, our nomogram excludes unimportant factors such as race and marital status, which helps doctors save time and effort in collecting unnecessary information.

There are limitations of the study. Firstly, this nomogram is constructed on the basis of retrospective cohort, and selection bias and recall bias may have influenced the results of our study. Prospective studies are required to validate our results. Secondly, the external validation cohort only comes from single center data, with a relatively small sample size. In the future, multicenter clinical trials with larger sample sizes and different ethnic groups are needed to evaluate the diagnostic performance of this prognostic model. Finally, the nomogram only includes clinicopathological characteristics collected from SEER database, and some potential predictive factors are not included. It is necessary to explore imaging and genetic factors related to the prognosis of IMPC patients with positive lymph nodes and further optimize the nomogram.

In summary, this study developed a nomogram to predict 3- and 5-year OS in lymph node-positive IMPC patients for the first time, and validation tests were carried out to confirm the reliability and accuracy of the developed models. The nomogram will help clinicians to stratify the risk of IMPC patients and develop personalized treatment strategies.

## Data availability statement

The raw data supporting the conclusions of this article will be made available by the authors, without undue reservation.

## Ethics statement

The studies involving humans were approved by The Ethics Review Committee of the Fourth Hospital of Hebei Medical University. The studies were conducted in accordance with the local legislation and institutional requirements. The participants provided their written informed consent to participate in this study.

## Author contributions

YFL, JL, and YPL designed the study. JS, SW and MZ extracted and analyzed the data. YFL and JL wrote and edited the manuscript. LFY and ZX reviewed and revised the manuscript. All authors contributed to the article and approved the submitted version.

## Supplementary material

Supplementary Figure 1 Histogram of the distribution of the training cohort data before and after Z-score normalization.

Supplementary Figure 2 Histogram of the distribution of the internal validation cohort data before and after Z-score normalization.

## References

[1] Siriaunkgul S Tavassoli FA. Invasive micropapillary carcinoma of the breast. Mod Pathol (1993) 6(6):660–2. 8302807

[2] Wang R Li N Wang XJ Chen T Zhang H Cheng YC. Differences in the clinicopathological characteristics of pure and mixed invasive micropapillary breast carcinomas from eastern China. Ann Transl Med (2021) 9(5):412. doi: 10.21037/atm-20-8045 33842633 PMC8033353

[3] Li W Han Y Wang C Guo X Shen B Liu F. Precise pathologic diagnosis and individualized treatment improve the outcomes of invasive micropapillary carcinoma of the breast: a 12-year prospective clinical study. Mod Pathol (2018) 31(6):956–64. doi: 10.1038/s41379-018-0024-8 29403084

[4] Kaya C Uçak R Bozkurt E Ömero ğ lu S Kartal K Yazici P. The impact of micropapillary component ratio on the prognosis of patients with invasive micropapillary breast carcinoma. J Invest Surg (2020) 33(1):31–9. doi: 10.1080/08941939.2018 29843540

[5] Fu L Ikuo M Fu XY Liu TH Shinichi T. Relationship between biologic behavior and morphologic features of invasive micropapillary carcinoma of the breast. Zhonghua Bing Li Xue Za Zhi (2004) 33(1):21–5. doi: 10.3760/j.issn:0529-5807.2004.01.006 14989923

[6] Guan X Xu G Shi A Zou Y Zhan Y Fan Z. Comparison of clinicopathological characteristics and prognosis among patients with pure invasive ductal carcinoma, invasive ductal carcinoma coexisted with invasive micropapillary carcinoma, and invasive ductal carcinoma coexisted with ductal carcinoma in situ: A retrospective cohort study. Med (Baltimore) (2020) 99(50):e23487. doi: 10.1097/MD.0000000000023487 PMC7738125 33327281

[7] Bansal GJ Jaipal A Wu GKC Syed A. Diagnostic accuracy of magnetic resonance imaging to evaluate axillary lymph node status in breast cancer patients receiving neoadjuvant chemotherapy. Br J Radiol (2023) 18:20220904. doi: 10.1259/bjr.20220904 PMC9975379 36607272

[8] Meng X Ma H Yin H Yin H Yu L Liu L. Nomogram predicting the risk of locoregional recurrence after mastectomy for invasive micropapillary carcinoma of the breast. Clin Breast Cancer (2021) 21(4):e368–76. doi: 10.1016/j.clbc.2020.12.003 33414079

[9] Chen AC Paulino AC Schwartz MR Rodriguez AA Bass BL Chang JC. Prognostic markers for invasive micropapillary carcinoma of the breast: a population-based analysis. Clin Breast Cancer (2013) 13(2):133–9. doi: 10.1016/j.clbc.2012.10.001 23246269

[10] Zhang Y Yu C. Development and validation of a Surveillance, Epidemiology, and End Results (SEER)-based prognostic nomogram for predicting survival in elderly patients with gastric cancer after surgery. J Gastrointest Oncol (2021) 12(2):278–96. doi: 10.21037/jgo-20-536 PMC8107591 34012626

[11] Chen Y Yu C Chen D Tang Y Zhu K Guo R. A prognostic nomogram based on risk assessment for invasive micropapillary carcinoma of the breast after surgery. Cancer Med (2023) 12(7):8050–62. doi: 10.1002/cam4.5595 PMC10134302 36602294

[12] Wang X Xue Y. Analysis of prognostic factors and construction of prognostic models for invasive micropapillary carcinoma of the breast. Comput Math Methods Med (2022) 2022:1072218. doi: 10.1155/2022/1072218 36339683 PMC9629958

[13] Hu C Shi F Zhang Z Zhang L Liu R Sun X. Development and validation of a new stage-specific nomogram model for predicting cancer-specific survival in patients in different stages of colon cancer: A SEER population-based study and external validation. Front Oncol (2022) 12:1024467. doi: 10.3389/fonc.2022.1024467 36568209 PMC9768485

[14] Dong Q Wu X Gan W Mok TN Shen J Zha Z. Construction and validation of web-based nomograms for detecting and prognosticating in prostate adenocarcinoma with bone metastasis. Sci Rep (2022) 12(1):18623. doi: 10.1038/s41598-022-23275-w 36329203 PMC9633700

[15] Tan X Wang J Tang J Tian X Jin L Li M. A nomogram for predicting cancer-specific survival in children with wilms tumor: A study based on SEER database and external validation in China. Front Public Health (2022) 10:829840. doi: 10.3389/fpubh.2022.829840 35462822 PMC9021525

[16] Plichta JK Ren Y Thomas SM Greenup RA Fayanju OM Rosenberger LH. Implications for breast cancer restaging based on the 8th edition AJCC staging manual. Ann Surg (2020) 271(1):169–76. doi: 10.1097/SLA.0000000000003071 PMC6588495 30312199

[17] Lewis GD Xing Y Haque W Patel T Schwartz M Chen A. Prognosis of lymphotropic invasive micropapillary breast carcinoma analyzed by using data from the National Cancer Database. Cancer Commun (Lond) (2019) 39(1):60. doi: 10.1186/s40880-019-0406-4 31639071 PMC6805396

[18] Chen AC Paulino AC Schwartz MR Rodriguez AA Bass BL Chang JC. Population-based comparison of prognostic factors in invasive micropapillary and invasive ductal carcinoma of the breast. Br J Cancer (2014) 111(3):619–22. doi: 10.1038/bjc.2014.301 PMC4119976 24921921

[19] Doll KM Rademaker A Sosa JA. Practical guide to surgical data sets: surveillance, epidemiology, and end results (SEER) database. JAMA Surg (2018) 153(6):588–9. doi: 10.1001/jamasurg.2018.0501 29617544

[20] Wang X Huang Z Li L Yang Y Zhang J Wang L. The role of alternative splicing factors, DDB2-related ageing and DNA damage repair in the progression and prognosis of stomach adenocarcinoma patients. Genes (Basel) (2022) 14(1):39. doi: 10.3390/genes14010039 36672781 PMC9858704

[21] Tibshirani R. Regression shrinkage and selection *via* the lasso. J R Stat Soc (Series B) (1996) 58:267–88. doi: 10.1111/j.2517-6161.1996.tb02080.x

[22] Hepp T Schmid M Gefeller O Waldmann E Mayr A. Approaches to regularized regression - A comparison between gradient boosting and the lasso. Methods Inf Med (2016) 55(5):422–30. doi: 10.3414/ME16-01-0033 27626931

[23] Luo B Yang M Han Z Que Z Luo T Tian J. Establishment of a nomogram-based prognostic model (LASSO-COX regression) for predicting progression-free survival of primary non-small cell lung cancer patients treated with adjuvant chinese herbal medicines therapy: A retrospective study of case series. Front Oncol (2022) 12:882278. doi: 10.3389/fonc.2022.882278 35875082 PMC9304868

[24] Wang P Wang L Liang X Si E Yang Y Kong L. Reconstructive types effect the prognosis of patients with tumors in the central and nipple portion of breast cancer? An analysis based on SEER database. Front Oncol (2023) 12:1092506. doi: 10.3389/fonc.2022.1092506 36755862 PMC9901204

[25] Fernandez G Prastawa M Madduri AS Scott R Marami B Shpalensky N. Development and validation of an AI-enabled digital breast cancer assay to predict early-stage breast cancer recurrence within 6 years. Breast Cancer Res (2022) 24(1):93. doi: 10.1186/s13058-022-01592-2 36539895 PMC9764637

[26] Dai X Cheng H Bai Z Li J. Breast cancer cell line classification and its relevance with breast tumor subtyping. J Cancer (2017) 8(16):3131–41. doi: 10.7150/jca.18457 PMC5665029 29158785

[27] Goto W Kashiwagi S Takada K Asano Y Takahashi K Fujita H. Significance of intrinsic breast cancer subtypes on the long-term prognosis after neoadjuvant chemotherapy. J Transl Med (2018) 16(1):307. doi: 10.1186/s12967-018-1679-0 30413161 PMC6230295

[28] Hwang KT Kim J Jung J Chang JH Chai YJ Oh SW. Impact of breast cancer subtypes on prognosis of women with operable invasive breast cancer: A population-based study using SEER database. Clin Cancer Res (2019) 25(6):1970–9. doi: 10.1158/1078-0432.CCR-18-2782 30559169

[29] Li Y Lu S Zhang Y Wang S Liu H. Loco-regional recurrence trend and prognosis in young women with breast cancer according to molecular subtypes: analysis of 1099 cases. World J Surg Oncol (2021) 19(1):113. doi: 10.1186/s12957-021-02214-5 33849563 PMC8042870

[30] Cosar R Sut N Ozen A Tastekin E Topaloglu S Cicin I. Breast cancer subtypes and prognosis: answers to subgroup classification questions, identifying the worst subgroup in our single-center series. Breast Cancer (Dove Med Press) (2022) 14:259–80. doi: 10.2147/BCTT.S380754 PMC9467695 36105268

[31] Lebwohl DE Canetta R. New developments in chemotherapy of advanced breast cancer. Ann Oncol (1999) 10(Suppl 6):139–46. doi: 10.1093/annonc/10.suppl_6.S139 10676565

[32] Kunkler IH Williams LJ Jack WJL Cameron DA Dixon JM. Breast-conserving surgery with or without irradiation in early breast cancer. N Engl J Med (2023) 388(7):585–94. doi: 10.1056/NEJMoa2207586 36791159

[33] Riaz N Jeen T Whelan TJ Nielsen TO. Recent advances in optimizing radiation therapy decisions in early invasive breast cancer. Cancers (Basel) (2023) 15(4):1260. doi: 10.3390/cancers15041260 36831598 PMC9954587

[34] Xu Y Zhang W He J Wang Y Chen R Shi W. Nomogram for predicting overall survival in patients with triple-negative apocrine breast cancer: Surveillance, epidemiology, and end results-based analysis. Breast (2022) 66:8–14. doi: 10.1016/j.breast.2022.08.011 36084385 PMC9465364

[35] Liu S Liu X Xiao Y Chen S Zhuang W. Prognostic factors associated with survival in patients with anaplastic oligodendroglioma. PloS One (2019) 14(1):e0211513. doi: 10.1371/journal.pone.0211513 30699183 PMC6353193

[36] Zhu Y Zhou C He Q. Radiation therapy's efficacy on tongue cancer: a population-based survival analysis. Onco Targets Ther (2018) 11:7271–6. doi: 10.2147/OTT.S169231 PMC6205818 30425518

[37] Yu JG Wu Z Ming Y Deng S Li Y Ou C. Prototypical multiple instance learning for predicting lymph node metastasis of breast cancer from whole-slide pathological images. Med Image Anal (2023) 85:102748. doi: 10.1016/j.media.2023.102748 36731274

[38] Ma D Yang Q Yin K Shi P Chen X Dong T. Analysis of the clinicopathological characteristics and prognosis of triple-positive breast cancer and HER2-positive breast cancer-A retrospective study. Front Oncol (2023) 12:999894. doi: 10.3389/fonc.2022.999894 36727058 PMC9885258

[39] Lai BS Tsang JY Li JJ Poon IK Tse GM. Anatomical site and size of sentinel lymph node metastasis predicted additional axillary tumour burden and breast cancer survival. Histopathology (2023) 82(6):899–911. doi: 10.1111/his.14875 36723261

