# Supplementary figures and images for: Construction and validation of a nomogram for predicting the prognosis of patients with lymph node-positive invasive micropapillary carcinoma of the breast: based on SEER database and external validation cohort
Source: Front Oncol. 2023 Oct 24;13:1231302. doi: 10.3389/fonc.2023.1231302 (PMC10635422; doi:10.3389/fonc.2023.1231302)

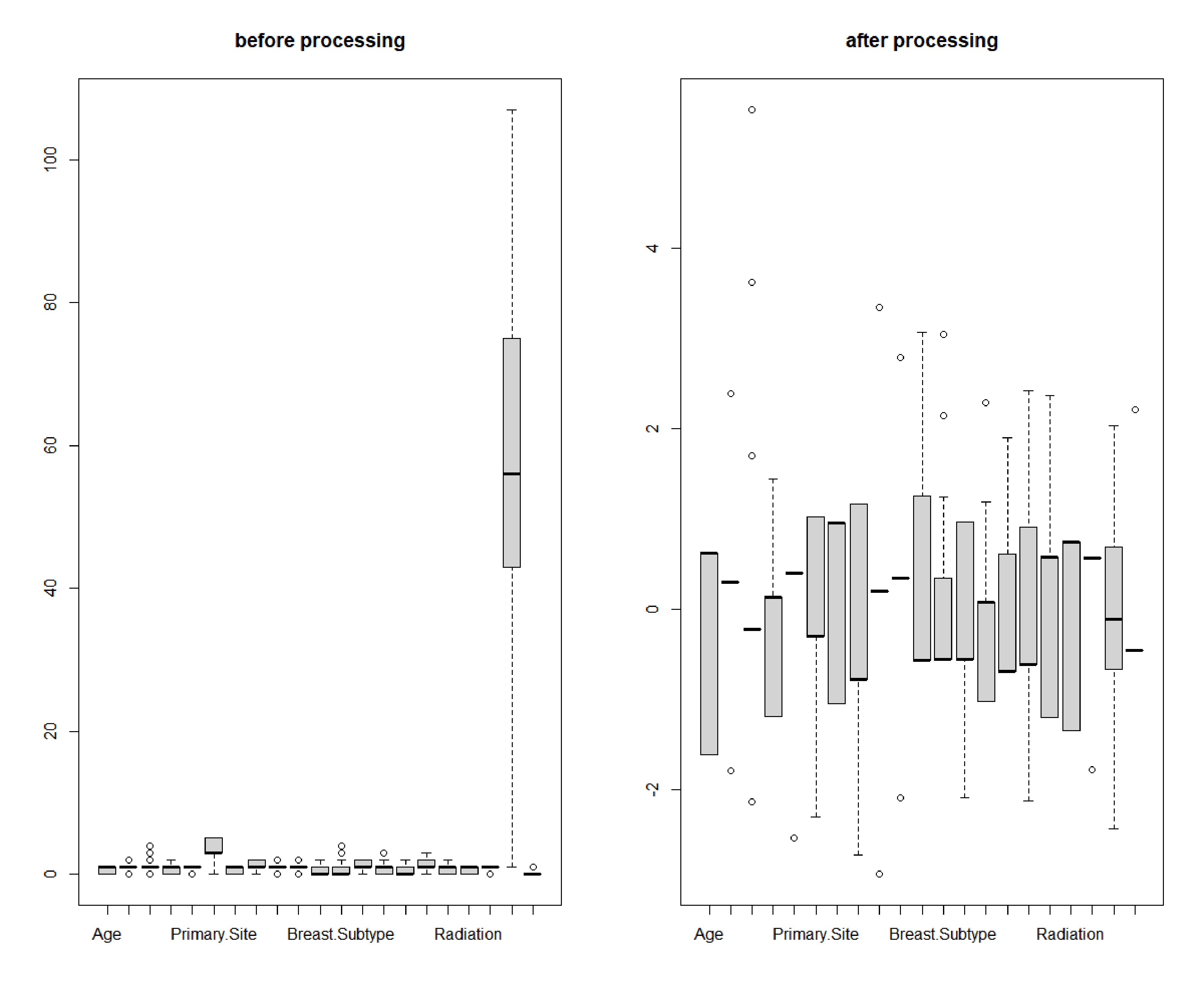

Supplement: Supplementary Figure 1 — Histogram of the distribution of the training cohort data before and after Z-score normalization. [file Image_1.jpeg]

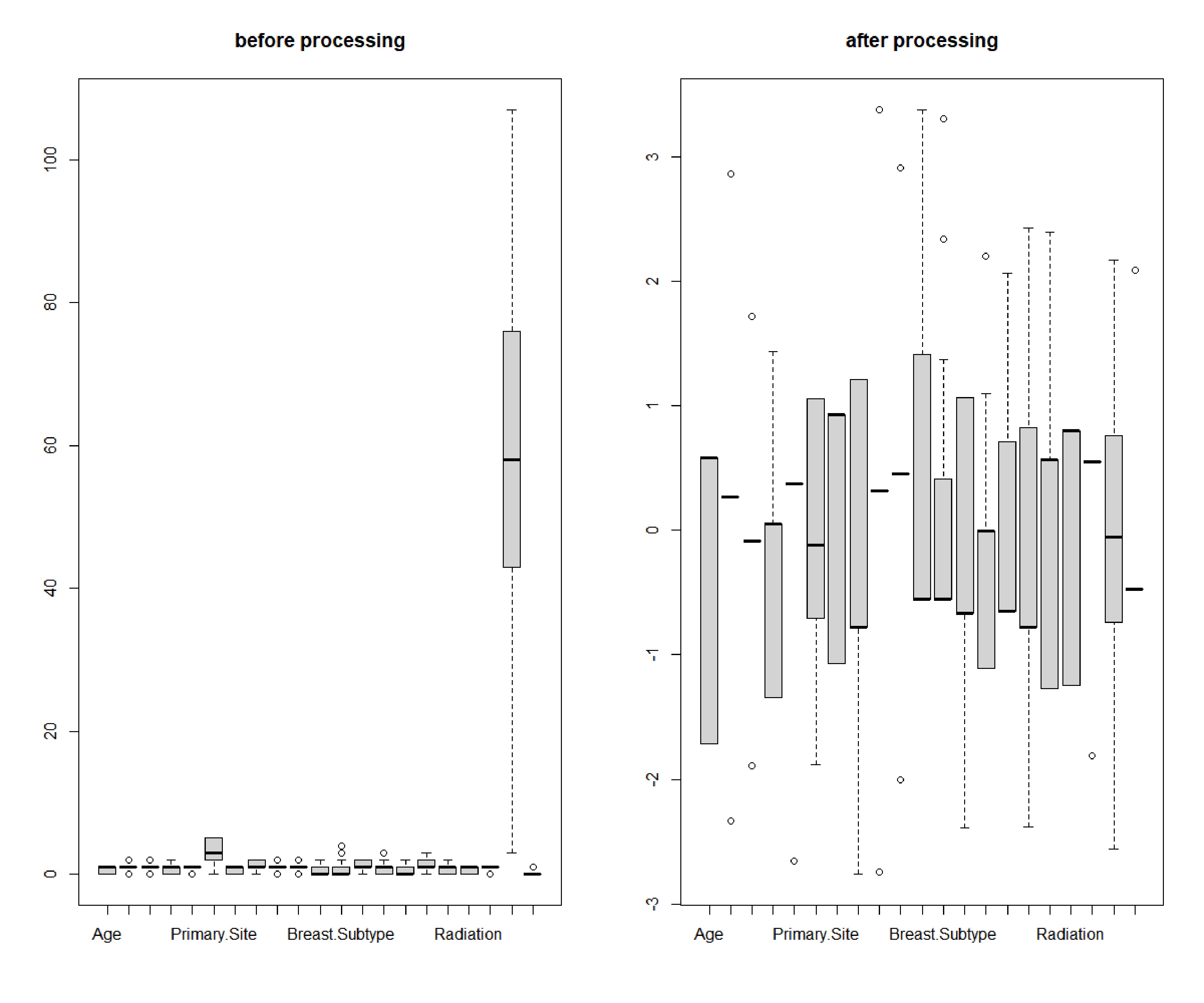

Supplement: Supplementary Figure 2 — Histogram of the distribution of the internal validation cohort data before and after Z-score normalization. [file Image_2.jpeg]
